# Isolation and Characterization of a Novel Lectin from the Edible Mushroom *Stropharia rugosoannulata*

**DOI:** 10.3390/molecules191219880

**Published:** 2014-11-28

**Authors:** Weiwei Zhang, Guoting Tian, Xueran Geng, Yongchang Zhao, Tzi Bun Ng, Liyan Zhao, Hexiang Wang

**Affiliations:** 1 State Key Laboratory for Agrobiotechnology and Department of Microbiology, China Agricultural University, Beijing 100193, China; E-Mails: zhangweiweicau@gmail.com (W.Z.); gengxueran2007@163.com (X.G.); 2 Institute of Biotechnology and Germplasmic Resource, Yunnan Academy of Agricultural Science, Kunming 650223, China; E-Mails: tiangt@aliyun.com (G.T.); yaasmushroom@aliyun.com (Y.Z.); 3 School of Biomedical Sciences, Faculty of Medicine, The Chinese University of Hong Kong, Shatin, New Territories, Hong Kong, China; E-Mail: b021770@mailserv.cuhk.edu.hk; 4 College of Food Science and Technology, Nanjing Agricultural University, Weigang, Nanjing 210095, China; E-Mail: zhlychen@njau.edu.cn

**Keywords:** lectin, *Stropharia rugosoannulata*, hemagglutinating activity, antiproliferative activity

## Abstract

To date, only a few steroids have been isolated from the mushroom *Stropharia rugosoannulata* which can be cultivated. In this paper, a novel lectin (SRL) with a molecular weight of 38 kDa, and a unique IKSGVYRIVSWQGALGPEAR N-terminal sequence was isolated from *S. rugosoannulata*, which represents the first protein isolated from the mushroom. The purification methods included (NH_4_)_2_SO_4_ precipitation, ion exchange chromatography on CM-cellulose, Q-Sepharose, and SP-Sepharose, and gel- filtration on Superdex-75. The lectin was adsorbed on all three types of ion exchangers and was purified more than 450-fold. The lectin was stable below 70 °C (with half of the activity preserved at 80 °C), and in the presence of NaOH and HCl solutions up to a concentration of 12.5 mM and 25 mM, respectively. The hemagglutinating activity of SRL was inhibited by inulin. Cd^2+^ and Hg^2+^ ions strongly reduced the hemagglutinating activity at concentrations from 1.25 mM to 10 mM. SRL exhibited anti-proliferative activity toward both hepatoma Hep G2 cells and leukemia L1210 cells, with an IC_50_ of 7 μM and 19 μM, respectively. The activity of HIV-1 reverse transcriptase could also be inhibited by SRL, with an IC_50_ of 10 μM.

## 1. Introduction

Lectins are proteins/glycoproteins which can agglutinate red blood cells with sugar specificity [1]. These proteins/glycoproteins have at least one non-catalytic domain that exhibits reversible binding to specific saccharides. Lectins were first isolated from plants [2,3], but are also found in various other organisms, including animals [4,5], bacteria [6] and fungi [7,8,9]. Lectins display an array of functions such as antifungal, antitumor, immunomodulatory, anti-insect, and antiviral activities. Hence, they have drawn the attention of many researchers. Fungi are a large repository of lectins in which 82% of the lectins identified are from mushrooms [10,11]. The biological roles of mushroom lectins encompass storage, growth, morphogenesis, parasitism, infections, molecular recognition, defense, cell flocculation, and mating [12,13]. To date, about 60 mushroom lectins have been identified and some of them were reported to possess antitumor, mitogenic, anti-HIV-1 reverse transcriptase [8] and immunoenhancing activities.

*Stropharia rugosoannulata*, which is called saketsubatake in Japanese and wine-cap stropharia in English, belongs to the Strophariaceae family which has a worldwide distribution in northern temperature zones. *S. rugosoannulata* is edible and can be cultivated for food. Some studies have been conducted on this species. A polysaccharide with potent antitumor and antioxidative effects has been purified [14]. Compounds with anti-endoplasmic-reticulum (ER) stress, anti-methicillin-resistant *Staphylococcus aureus* (MRSA), anti-fungal, and osteoclast formation suppressing activities have been isolated [15,16]. Besides, bioactive steroids in *S. rugosoannulata* were found to have an effect on lettuce growth [17]. However, there are no reports on proteinaceous constituents. The objective of the present study was to isolate a lectin from *S. rugosoannulata*, and compare its biological and physicochemical characteristics with other mushroom lectins to thus see if this lectin possesses potentially exploitable activities.

## 2. Results and Discussion

### 2.1. Results

**Figure 1:**
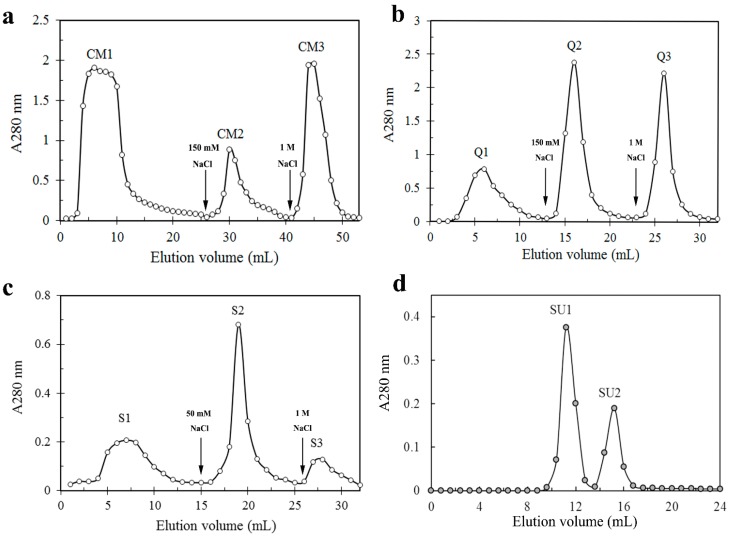
Purification of *S. rugosoannulata* lectin (SRL) by chromatography on (**a**) CM-celluose (2.5 cm × 30 cm); (**b**) Q-Sepharose (1 cm × 10 cm); (**c**) SP-Sepharose (1 cm × 10 cm) and (**d**) gel-filtration on Superdex G-75 HR10/30 column. Arrows indicate the point at which buffer was changed.

The extract of the *Stropharia rugosoannulata* fruiting bodies was pretreated by precipitation by 70% ammonium sulfate. We obtained three fractions (CM 1–3) after loading the sample, which had been dialyzed against sodium acetate buffer (pH5.2), on a CM-cellulose chromatography column, and the lectin activity was detected in fraction CM2 (Figure 1a). Fraction CM2 was then subjected to chromatography on Q-Sepharose and lectin activities was enriched in the second (Q2) of the three resulting fractions Q1-3 (Figure 1b). Subsequently, fraction Q2 was then separated into three fractions S1–S3 by SP-sepharose chromatography. The lectin activity resided in fraction S2 (Figure 1c). Upon gel filtration on Superdex 75, S2 was resolved into a large peak SU1 and a small peak SU2 (Figure 1d). Hemagglutinating activity was confined to SU1, which possessed a molecular mass of 36 kDa.

**Figure 2:**
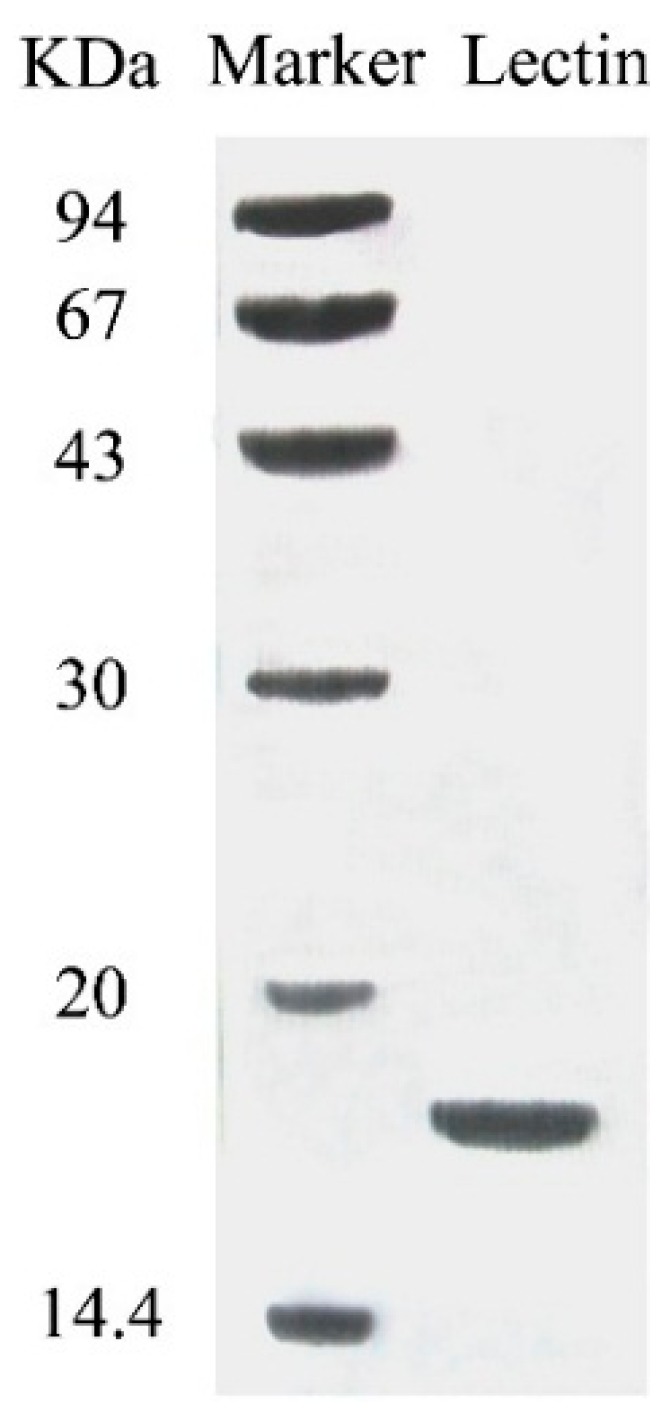
SDS-PAGE results of fraction SU1 which represents purified SRL.

The purified lectin appeared as a single band with a molecular mass of 18 kDa in SDS-PAGE (Figure 2). This suggests that the lectin was composed of two subunits, each with a molecular mass of 18 kDa. The N-terminal sequence of this lectin was IKSGVYRIVSWQGALGPEAR. A BLAST search did not reveal sequence resemblance to any previously published mushroom lectin or other lectins.

The yields and specific hemagglutinating activities of chromatographic fractions are given in Table 1 which shows that the lectin was purified more than 450-fold. The hemagglutinating activity of SRL remained stable between 10 °C and 70 °C, while 50% activity remained at 80 °C and 25% activity was left at 90 °C. No activity was detectable at 100 °C (Table 2). The activity was retained in the presence of 6 mM and 12.5 mM HCl (pH 1.9) and 6 mM NaOH (pH 11.7), but it was only 50% when the HCl concentration was increased to 25 mM (pH 1.6) and that of NaOH to 12.5 mM (pH 12.1). There was no activity detectable at 100 mM NaOH or HCl concentration (pH below 1 and above 13, respectively) (Table 3).

**Table 1:** Summary of purification of *S. rugosoannulata* lectin (from 400 g fresh fruiting bodies).

Fraction	Yield (mg)	Specific Activity (U/mg)	Total Activity (U)	Recovery of Activity (%)	Folds of Purification
CM	11,203.8	0.7	8105.0	100.0	1.0
CM1	2125.7	-	-	-	-
CM2	280.5	11.3	3170.6	39.1	16.1
CM3	1286.3	-	-	-	-
Q1	51.	-	-	-	-
Q2	44.6	44.4	1978.2	24.4	63.4
Q3	46.9	-	-	-	-
S1	11.9	-	-	-	-
S2	12.3	117.8	1453.6	17.9	168.3
S3	5.6	-	-	-	-
SU1	2.0	320.0	640.0	7.9	457.1
SU2	1.2	-	-	-	-

**Table 2:** Effects of temperature on hemagglutinating activity of *S. rugosoannulata* lectin. (initial hemagglutinating activity: 64U). *S. rugosoannulata* lectin was incubated at different temperatures for 30 min before determination of hemagglutinating activity.

Temperature (°C)	10	20	30	40	50	60	70	80	90	100
Hemagglutinating activity (U)	64	64	64	64	64	64	32	16	16	0

**Table 3:** Effects of NaOH and HCl solutions on hemagglutinating activity of *S. rugosoannulata* lectin. (initial hemagglutinating activity: 64U). *S. rugosoannulata* lectin was incubated at different pH values for 60 min before determination of hemagglutinating activity.

The Hemagglutinating Activity of SRL in Different Concentrations of HCl						
HCl concentration (M)	6 (pH 2.2)	12.5 (pH 1.9)	25 (pH 1.6)	50 (pH 1.3)	100 (pH 1.0)	200 (pH 0.7)
Hemagglutinating Activity (U)	64	64	32	8	0	0
**The Hemagglutinating Activity of SRL in Different Concentrations of NaOH**						
NaOH concentration (M)	6 (pH 11.7)	12.5 (pH 12.1)	25 (pH 12.4)	50 (pH 12.7)	100 (pH 13.0)	200 (pH 13.3)
Hemagglutinating Activity (U)	64	32	16	8	0	0

The hemagglutinating activity of the lectin was not affected by the majority of divalent and trivalent metallic ions tested (Fe^2+^, Cu^2+^, Al^3+^, Mg^2+^, and K^+^). Zn^2+^, Ca^2+^, and Mg^2+^ ions at a higher concentration (10 mM) slightly affected the hemagglutinating activity. The activity was drastically reduced by Cd^2+^ and Hg^2+^ ions at a concentration range of 1.25 mM to 10 mM, and dose-dependently reduced by Pb^2+^ and Fe^3+^ ions (Table 4).

**Table 4:** Effects of cations on hemagglutinating activity of *S. rugosoannulata* lectin. (initial hemagglutinating activity: 64U). *S. rugosoannulata* lectin was incubated in presence of different metal ions for 60 min before determination of hemagglutinating activity.

Cations	10 mM	5 mM	2.5 mM	1.25 mM
Cd^2+^	0	0	2	4
Fe^3+^	64	64	64	64
Cu^2+^	64	64	64	64
Hg^2+^	0	0	2	4
Al^3+^	64	64	64	64
Pb^2+^	4	16	32	64
Zn^2+^	32	32	64	64
Ca^2+^	32	64	64	64
Mn^2+^	32	64	64	64
Mg^2+^	64	64	64	64
K^+^	64	64	64	64
Fe^2+^	0	4	16	64

All sugars tested excepted inulin had no effect on the hemagglutinating activity of the lectin when tested at various concentrations from 0.87 mM to 200 mM. The minimum concentration which inhibited the hemagglutinating of inulin was 50 mM (Table 5).

**Table 5:** Effects of various carbohydrates on hemagglutinating induced by *S. rugosoannulata* lectin.

Sugar	Minimum Inhibitory Concentration of Sugar (mM)
l-Sorbose	No
Raffinose	No
l-Rhamnose	No
d-Fructose	No
d-Mannose	No
Cellobiose	No
l-Arabinose	No
d-Xylose	No
d-Melibiose	No
Lactose	No
Inulin	0.1 M
Maltose	No
d-Galactose	No
d-Glucose	No

**Figure 3:**
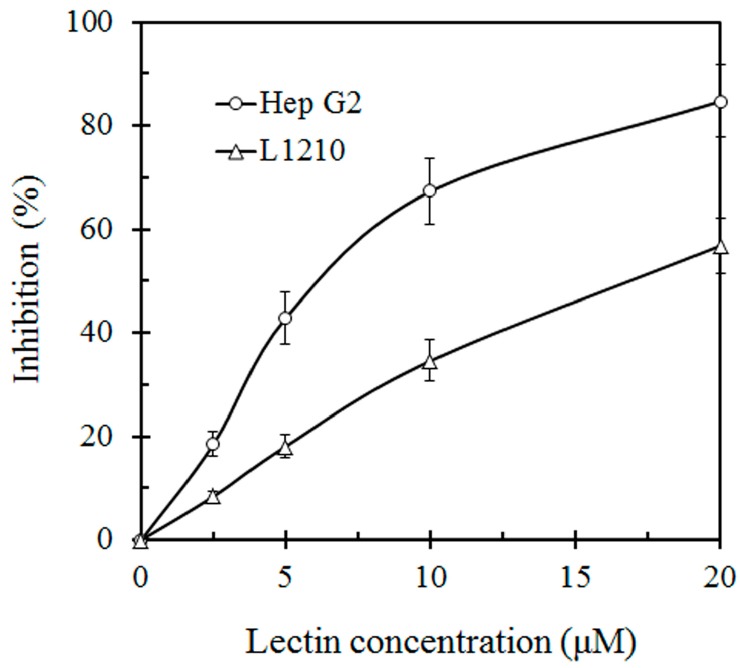
*In vitro* inhibitory effect of *S.* *rugosoannulata* lectin (SRL) on proliferation of hepatoma Hep G2 cells and murine leukemia L1210 cells. Proliferation of HepG2 cells and L1210 cells was inhibited with an IC_50_ of 7 μM and 19 μM, respectively.

**Figure 4:**
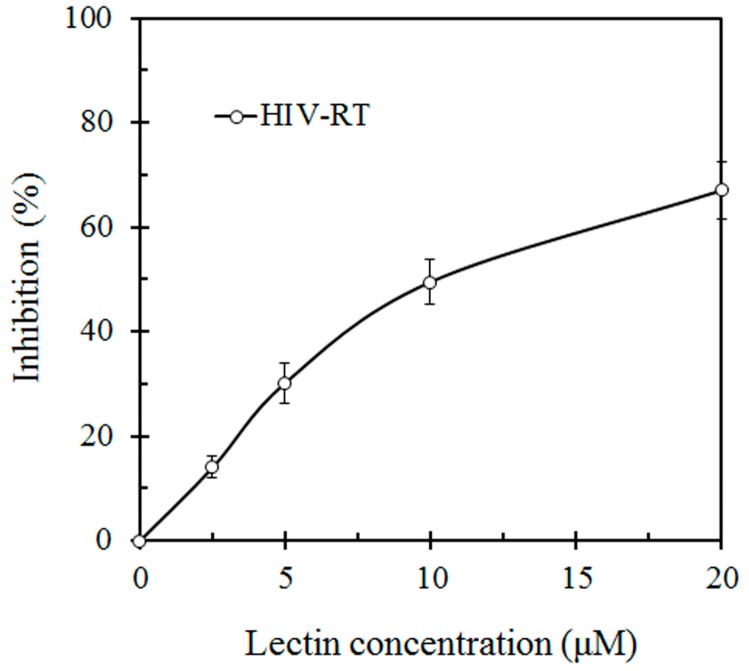
Inhibitory effect of *S.* *rugosoannulata* lectin (SRL) on HIV-1 reverse transcriptase (HIV-RT). HIV-RT activity was inhibited with an IC_50_ of 10 μM.

This lectin inhibited the proliferation of HepG2 and L1210 tumor cells with an IC_50_ value of 7 μM and 19 μM, respectively (Figure 3). SRL also had anti HIV-1 reverse transcriptase activity with an IC_50_ value of 10 μM (Figure 4).

### 2.2. Discussion

*Stropharia rugosoannulata* is an edible mushroom which can be cultivated for food. In the genus *Stropharia*, *S. rugosoannulata* and *Stropharia aeruginosa* are the two species which are relatively better known than others. However, few studies about either of them have been reported. In this study, we isolated a lectin (SRL) from *S. rugosoannulata* which represents the first protein purified from this species. The finding of SRL may promote the development of *S. rugosoannulata* to some degree.

SRL has been purified more than 450-fold in this study, which is much higher than the values for other lectins [7,18]. During the purification procedure, four chromatographic steps including three ion exchange columns and one gel-filtration step were involved. SRL is not adsorbed on DEAE-cellulose, but is adsorbed on CM-cellulose, Q-sepharose, and SP-sepharose.

The molecular weight determined by SDS-PAGE is half of the amount determined by gel-filtration chromatography. This suggests that SRL is dimeric, like lectins from other mushrooms including *Pleurotus citrinopileatus* [8] and *Agrocybe cylindracea* [19]. The molecular mass of SRL is within the range shown by mushroom lectins [20].

SRL is the first protein isolated from *Stropharia rugosoannulata* with a unique *N*-terminal sequence. Little resemblance of this *N*-teminal sequence can be found between SRL and other mushroom lectins, such as those from *Agaricus bisporus* [21], *Agrocybe aegerita* [22], *Coprinopsis cinerea* [23], *Flammulina velutipes* [24], *Laccaria bicolor* [25], and *Pleurotus cornucopiae* [26] (shown in Table 6).

**Table 6:** Comparison of N-terminal sequence of *S. rugosoannulata* lectin with sequences of known mushroom lectins.

Species	N-Terminal Sequence
*Stropharia rugosoannulata*	IKSGVYRIVSWQGALGPEAR
*Agaricus bisporus* [21]	MGGSGTSGSL
*Agrocybe aegerita* [22]	NISAGTSVDL
*Coprinopsis cinerea* [23]	IPLEGTFGDR
*Flammulina velutipes* [24]	TSLTFQLAYL
*Laccaria bicolor* [25]	SHLYGDGVAL
*Pleurotus cornucopiae* [26]	SDSTWTFAML

Compared with other lectins isolated from mushrooms, SRL has advantages in its possession of anti-proliferative activity toward cancer cells and anti-HIV-RT activity. SRL is shown to have an ability to inhibit proliferation of two tumor cell lines, Hep G2 and L1210. The potent antiproliferative activity of SRL could facilitate its development into a cancer therapy agent. As a key enzyme of the HIV-1 life cycle, HIV-1 RT is a target of anti-HIV-1 drugs. It is worth mentioning that SRL manifests potent inhibitory activity towards HIV-1 RT with an IC_50_ of 10 μM.

SRL is moderately thermostable. Its hemagglutinating activitity is not affected below 70 °C. At 80 °C, 50% of the activity remains. It is thus more thermostable than lectins from *Boletus edulis* [27,28] and some other mushrooms, but similar to lectins from *Russula lepida* [9] and *Tricholoma mongolicum* [29]. SRL is stable in the presence of 0.05 M NaOH and 0.1 M HCl solution. Hemagglutinating activity is detectable even in 0.2 M NaOH and HCl. In this aspect, SRL is more stable than some other mushroom lectins [18,28,30].

The lectin activity of SRL remains unaltered after metal ion chelation with EDTA or in the presence of various cations , like Fe^2+^, Cu^2+^, Al^3+^, Mn^2+^ and K^+^ ions, and is slightly inhibited in the presence of high concentrations of Zn^2+^ and Ca^2+^ ions. The hemagglutinating activity of SRL is strongly inhibited by Cd^2+^ and Hg^2+^ ions. The fact that no increase in activity be detected in the presence of the metal ions suggests that the active region of SRL may not contain any metal ions. The inhibitory effect of Cd^2+^ and Hg^2+^ ions indicates that SRL hemagglutinating activity may be reduced by heavy metals, probably due to protein denaturation.

Lectin is the marker for blood grouping. However, SRL cannot agglutinate human erythrocytes of any of the groups of the ABO system. Besides, SRL can agglutinate rabbit red blood cells. This probably suggests SRL cannot bind the SRL receptor of human erythrocytes but has a specific binding with rabbit erythrocytes. However, among the sugars involved in the test of carbohydrate specificity, only inulin was found to have inhibition effect to hemagglutinating activity of SRL. Inulin is a plant polysaccharide, which is a heterogeneous collection of fructose polymers. Lectins from plants and some mushroom specifically recognize simple sugars and disaccharides. However, there are a few lectins isolated from mushrooms whose hemagglutinating activity could only be inhibited by inulin [31,32]. To date, only few isolated lectins are found to be inulin-specific [18,30,31]. Hence SRL may have a potential to be used in carbohydrate binding research and affinity chromatography.

## 3. Experimental Section

### 3.1. Isolation of Lectins

Fresh fruiting bodies of *Stropharia rugosoannulata* were purchased from a local market in Dandong, Liaoning Province, China. *S. rugosoannulata* (400 g) were homogenized in 0.15 M NaCl at 4 °C and extracted overnight. The homogenate was centrifuged at 9000 rpm for 20 min at 4 °C. The supernatant was added to 80% (NH_4_)_2_SO_4_ and stirred. The precipitate was collected by centrifugation at 9000 rpm for 20 min, dissolved in and dialyzed against distilled water and dialyzed extensively before applying on a CM-cellulose (Sigma, St. Louis, MO, USA) column (2.5 cm × 20 cm) which had previously been equilibrated with 10 mM phosphate buffer (pH 5.2). After removal of the unadsorbed fraction C1, the adsorbed fractions C2 and C3 were eluted with 0.15 M and 1 M NaCl in phosphate buffer. Hemagglutinating activity was enriched in fraction C2. Fraction C2 was then subjected to ion exchange chromatography on a 1.0 cm × 10 cm column of Q-Sepharose (GE Healthcare, Uppsala, Sweden) in 10 mM NaOAc buffer (pH 6.1). Three fractions Q1, Q2 and Q3 were collected after elution with 0.05 M, 0.15 M and 1 M NaCl in 10 mM NaOAc buffer (pH 6.1), respectively. The hemagglutinating activity was enriched in the Q2. Peak Q2 was then applied on an SP-Sepharose (GE Healthcare) (1.0 cm × 10 cm) column in 10 mM NaOAc buffer (pH 5.0). Unbound material was eluted with the starting buffer while bound material was desorbed by addition of 50 mM NaCl, and 1 M NaCl in the starting buffer, respectively. The active peak (S2) containing hemagglutinating activity was subsequently subjected to gel filtration by fast protein liquid chromatography on a Superdex75 HR 10/30 column (GE Healthcare) in 0.15 M NH_4_HCO_3_ buffer (pH 8.5) using an AKTA Purifier (GE Healthcare). The first peak (SU1) constituted the purified lectin.

### 3.2. Determination of Molecular Mass and N-Terminal Sequence

For molecular mass determination, the purified lectin was subjected to sodium dodecyl sulfate-polyacrylamide gel electrophoresis (SDS-PAGE) in accordance with the procedure of Laemmli and Favre [32]. The molecular mass of the purified protein was also determined by FPLC-gel filtration as described above. A Hewlett-Packard HP G1000A Edman degradation unit and an HP 1000 HPLC System (GE Healthcare) were used for the *N*-terminal sequence determination.

### 3.3. Assay for Hemagglutinating Activity

A serial two-fold dilution of the lectin solution in microtiter U-plates (25 μL) was mixed with 25 μL of a 2% suspension of rabbit red blood cells in phosphate-buffered saline (pH 7.2) at 20 °C. The results were read after about 1 h when the blank had fully sedimented. The hemagglutination titer, defined as the reciprocal of the highest dilution of the lectin solution exhibiting hemagglutination, was reckoned as one hemagglutination unit. Specific activity is the number of hemagglutination units per mg protein.

To test the inhibition of lectin-induced hemagglutination by various carbohydrates, hemagglutinating inhibition tests were performed in a manner analogous to the hemagglutination test. l-Sorbose, raffinose, l-rhamnose, d-fructose, d-mannose, cellobiose, l-arabinose, d-xylose, d-melibiose, lactose, inulin, maltose, d-galactose, and d-glucose were included in the carbohydrates tested. Serial two-fold dilutions of sugar samples were prepared in phosphate-buffered saline. An equal volume (25 μL) of a solution of the lectin with eight hemagglutination units was mixed with all the dilutions. The mixture was allowed to stay at room temperature for 1 h, and then mixed with 50 μL of 2% rabbit erythrocyte suspension. The minimum concentration of the sugar in the final mixture, which completely inhibited eight hemagglutination units of the lectin preparation, was determined [18].

The effects of temperature, NaOH solution and HCl solution, and solutions of metallic chlorides on hemagglutinating activity of 64 hemagglutinating units of purified lectin after exposure for 30 or 60 min was examined as previously described [28].

### 3.4. Assay of Antiproliferative Activity on Tumor Cell Lines

The antiproliferative activity of the purified lectin was determined as follows. Two cell lines including the hepatoma cell lines HepG2 and murine leukemia L1210 cells lines purchased from American Type Culture Collection (ATCC, Manassas, VA, USA) were tested. HepG2 cells were maintained in Dulbecco modified Eagles’ Medium (DMEM) while L1210 cells were maintained in Roswell Park Memorial Institute 1640 (RPMI-1640) supplemented with 10% fetal bovine serum (FBS) and 100 mg/L streptomycin and 100 IU/mL penicillin. Both were cultivated at 37 °C in a humidified atmosphere of 5% CO_2_. Cells (1 × 10^4^) in their exponential growth phase were seeded into each well of a 96-well culture plate and incubated for 3 h before addition of the lectin. Incubation was carried out for another 48 h. Radioactive precursor, 1 μCi, ([^3^H-methyl] thymidine, from GE Healthcare) was then added to each well and incubated for 6 h. The cultures were then harvested by a cell harvester. The incorporated radioactivity was determined by liquid scintillation counting.

### 3.5. Assay for HIV-1 Reverse Transcriptase Inhibitory Activity

The assay was carried out according to instructions supplied with the assay kit from Boehringer Mannhein (Columbus, OH, USA). The assay takes advantage of the ability of reverse transcriptase to synthesize DNA, starting from the template/primer hybrid poly (A) oligo (dT). The digoxigenin- and biotin-labeled nucleotides in an optimized ratio are incorporated into one of the same DNA molecule, which is freshly synthesized by the reverse transcriptase (RT). The detection and quantification of synthesized DNA as a parameter for RT activity follows a sandwich ELISA protocol. The absorbance of the samples at 405 nm can be determined using a microtiter plate (ELISA) reader and is directly correlateed to the level of RT activity. A fixed amount (4–6 ng) of recombinant HIV-1 reverse transcriptase was used. The inhibitory activity of the lectin was calculated as percent inhibition as compared to a control without the protein.

## 4. Conclusions

In summary, a lectin with a distinctive N-terminal sequence, carbohydrate specificity, relatively high thermostability and potent antiproliferative activity was purified from *S. rugosoannulata* fruiting bodies. This report represents the first lectin isolated from the mushroom belonging to the Stropharia genus. In view of the very few publications on proteins from *Stropharia* species, the present article is a valuable addition to this scanty literature. 
